# AI-driven drug discovery and repurposing using multi-omics for myocardial infarction and heart failure

**DOI:** 10.37349/emed.2025.1001340

**Published:** 2025-06-25

**Authors:** Ziad Sabry, Harkirat Singh Arora, Sriram Chandrasekaran, Zhong Wang

**Affiliations:** 1 Cellular and Molecular Biology Program, University of Michigan, Ann Arbor, MI 48109, USA; 2 Gilbert S. Omenn Department of Computational Medicine and Bioinformatics, Ann Arbor, MI 48109, USA; 3 Department of Biomedical Engineering, University of Michigan, Ann Arbor, MI 48109, USA; 4 Program in Chemical Biology, University of Michigan, Ann Arbor, MI 48109, USA; 5 Department of Cardiac Surgery, Frankel Cardiovascular Center, University of Michigan, Ann Arbor, MI 48109, USA

**Keywords:** Explainable artificial intelligence, heart failure, drug discovery, drug repurposing

## Abstract

Cardiovascular diseases (CVDs) are the leading causes of morbidity and mortality worldwide. Yet, drug discovery for these conditions faces significant challenges due to the complexity and heterogeneity of their underlying pathology. Recently, artificial intelligence (AI) techniques—particularly explainable AI (XAI)—have emerged as powerful multi-omics data analyzing tools to unravel pathological mechanisms and novel therapeutic targets. However, the application of XAI in cardiovascular drug discovery remains in its infancy. This review discusses the potential for the integration of AI with multi-omics data to identify novel therapeutic targets and repurpose existing drugs for myocardial infarction (MI) and heart failure (HF). This review highlights the current gap in leveraging XAI for CVDs and discusses key challenges such as data heterogeneity, model interpretability, and translational validation. This review also describes emerging approaches, including combining AI with mechanistic models, that aim to enhance the biological relevance of AI predictions. By utilizing genomic, transcriptomic, epigenomic, proteomic, and metabolomic datasets, AI-driven methods can uncover new biomarkers and predict drug responses with greater precision. The application of AI in analyzing large-scale clinical and molecular data offers significant promise in accelerating drug discovery, refining therapeutic strategies, and improving outcomes for patients with CVDs. This review highlights recent advancements, challenges, and future directions for AI-guided drug discovery in the context of MI and HF.

## Introduction

Cardiovascular diseases (CVDs) represent significant health burdens, contributing to high mortality rates globally [1]. Despite extensive research into CVDs, the complexity of their molecular mechanisms presents substantial challenges to drug discovery efforts. Traditional approaches have been limited by the heterogeneity of these conditions, low tolerance of adverse effects, and the long timelines associated with developing novel therapeutics [2, 3]. In recent years, advancements in bioinformatics and artificial intelligence (AI) have opened new avenues for addressing these challenges. AI, particularly explainable AI (XAI), enables researchers to interpret large, complex datasets and identify patterns that may not be evident through conventional methods. Recent reviews highlight how AI, in combination with biosensing technologies and systems biology approaches, is reshaping the landscape of disease detection through bioimaging across multiple disease areas, including oncology, neurology, and infectious diseases [4].

However, the adoption of AI—especially XAI—for cardiovascular drug discovery has lagged behind its impact in fields like oncology and neurology [5]. Only a small fraction of XAI studies to date have focused on heart disease, highlighting a critical gap in this domain. We have therefore chosen to focus this review on myocardial infarction (MI) and heart failure (HF), two CVD conditions that are major contributors to morbidity and mortality and where new therapies are urgently needed. These conditions represent acute MI and chronic HF cardiac pathologies for which multi-omics datasets are increasingly available, making them prime candidates for AI-driven investigation.

This focuses on the integration of AI with multi-omics data to uncover mechanisms of MI and HF, identify therapeutic targets, and advance drug repurposing. The transformative potential of AI in revolutionizing drug discovery for MI and HF lies in its ability to integrate multi-omics data, including genomic, transcriptomic, epigenomic, proteomic, and metabolomic datasets. By leveraging these comprehensive datasets, AI can elucidate the pathological mechanisms underlying MI and HF, identify novel therapeutic targets, and predict drug response with greater precision. Moreover, AI-driven approaches to drug repurposing, which aim to identify new uses for existing FDA-approved drugs, have gained significant attention as a cost-effective strategy for accelerating therapeutic development. This review focuses on the intersection of AI, bioinformatics, and drug discovery in MI and HF while drawing comparisons to advancements in fields such as oncology and neurology, where AI-driven research is more established. By extrapolating insights from these advanced areas, we outline future directions for AI-enabled cardiovascular research. Ultimately, AI has the potential to redefine cardiovascular medicine, paving the way for innovative treatments and improved patient outcomes.

## Methodology

**Figure 1.**
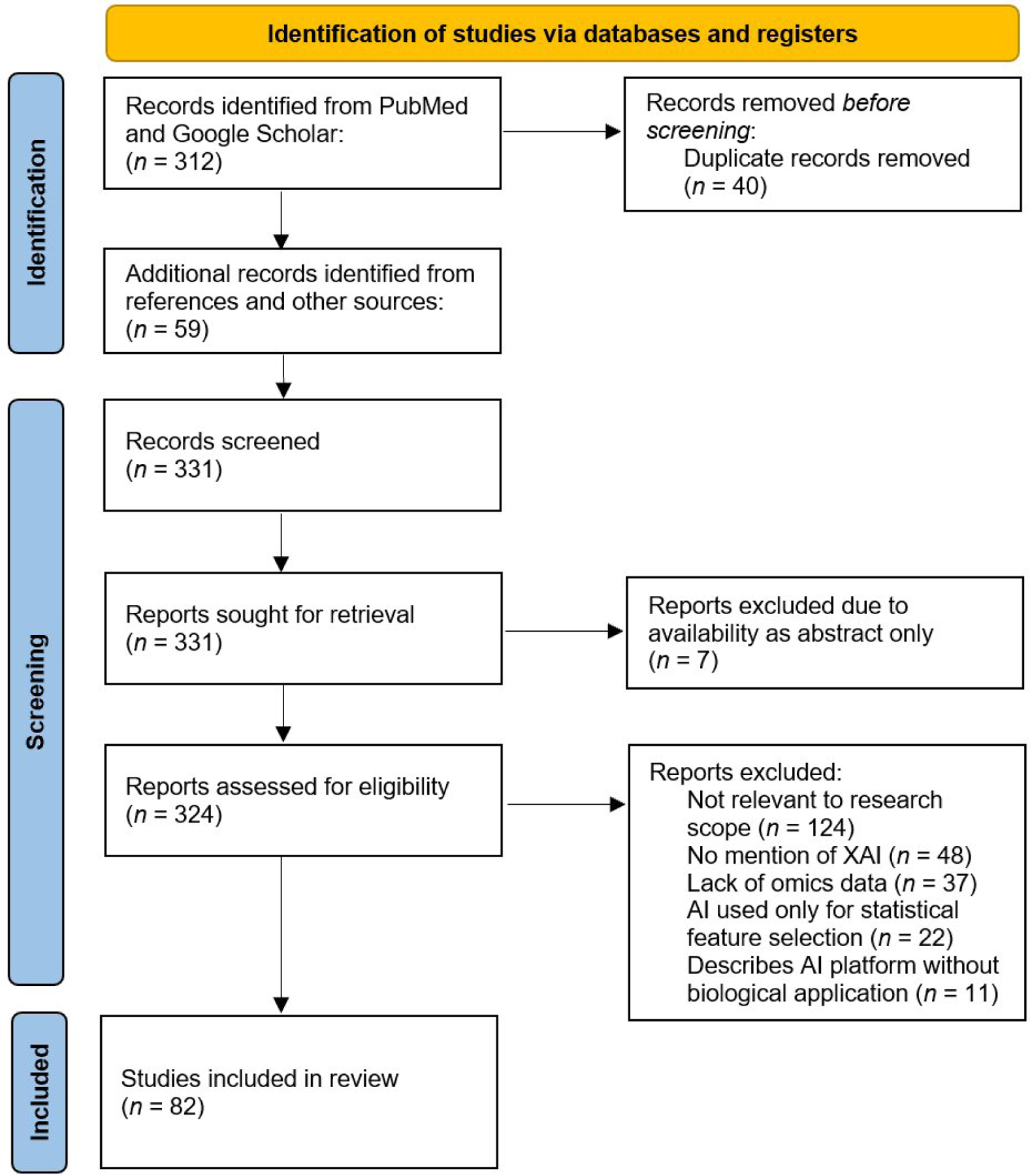
PRISMA flow diagram summarizing the study selection process for inclusion in this review. The literature review was conducted in accordance with PRISMA guidelines. XAI: explainable artificial intelligence. Adapted from [6], CC BY

This literature review was conducted in accordance with the PRISMA guidelines to ensure a transparent and reproducible methodology [6]. We performed comprehensive searches of publication databases (including PubMed and Google Scholar) up to January 2025 for English-language articles focused on AI or XAI applications in oncology, neurology, infectious diseases, and CVDs, particularly MI and HF. Key search terms included combinations of “artificial intelligence”, “machine learning”, “explainable AI”, “XAI”, “multi-omics”, “oncology”, “neurology”, “infectious disease”, “myocardial infarction”, “heart failure”, “drug discovery”, and “drug repurposing”. Relevant studies were selected based on their focus on AI-driven analysis of molecular (omics) data or drug repurposing in the disease context. Reference lists of pertinent articles were also reviewed to identify additional reports. The PRISMA flow diagram (Figure 1) illustrates the article selection process, including the number of records identified, screened, excluded, and included.

Within this review, we categorize AI-driven techniques into traditional machine learning (ML) methods (e.g., random forests, support vector machines), deep learning approaches (e.g., convolutional or graph neural networks), and hybrid AI methods that incorporate biological knowledge. We also highlight prominent AI model interpretation techniques such as feature importance ranking and model visualization [e.g., SHapley Additive exPlanations (SHAP), Local Interpretable Model-agnostic Explanations (LIME), attention maps], as well as emerging strategies that combine AI with mechanistic modeling. These methodological considerations establish the foundation for the results and discussions presented in the subsequent sections.

## Omics data as a foundation for AI study in cardiovascular medicine

**Figure 2.**
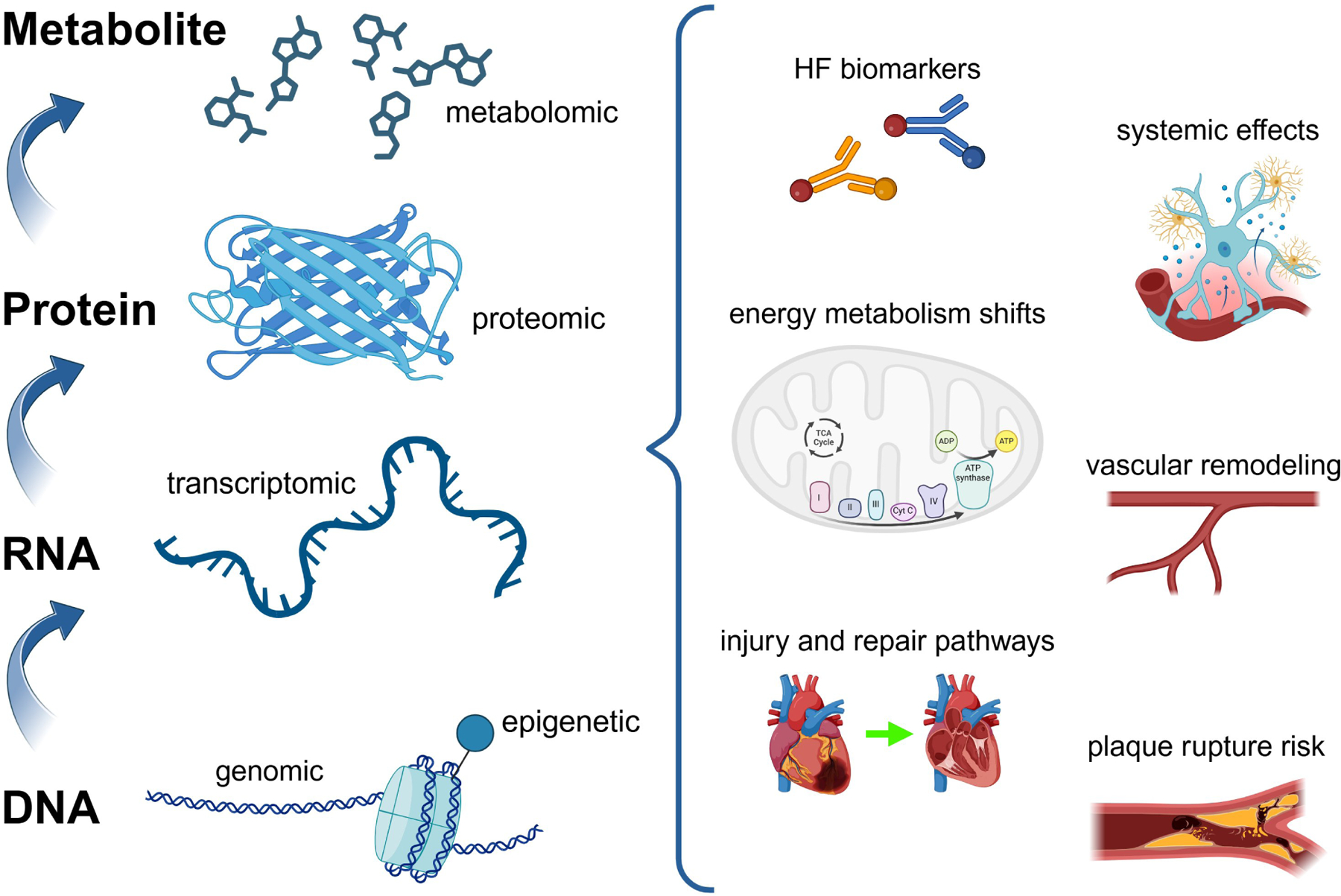
Overview of multi-omics data as the foundation for AI in studying cardiac biology. From bottom to top: genomic data provides insights into the DNA sequence and variations; epigenetic data reflects chromatin modifications and regulatory changes; transcriptomic data captures RNA profiles and gene expression patterns; proteomic data characterizes protein structure, function, and interactions; metabolomic data reveals cellular metabolic states. The multi-omics layers on the left side of the figure collectively identify key pathways, metabolic changes, and protein interactions, which are depicted on the right side as critical components of cardiac biology and disease mechanisms. AI: artificial intelligence; HF: heart failure; TCA: tricarboxylic acid; Cyt C: cytochrome C. Created in BioRender. Sabry, Z. (2025) https://BioRender.com/t20l803

The heart is a remarkably specialized organ composed of intricate cell types such as cardiomyocytes, smooth muscle cells, and endothelial cells. Each of these cell types possesses unique chromatin structures, metabolic profiles, transcriptomes, and proteomes, reflecting their complex biological roles. With the advent of high-throughput technologies, researchers now have access to vast datasets across multiple biological layers, including genomic, transcriptomic, epigenetic, proteomic, and metabolomic data (Figure 2). These datasets collectively offer a systems-level understanding of the heart’s molecular landscape, enabling the detailed characterization of cell-specific functions, molecular interactions, and their contributions to cardiac physiology and pathology. However, fully integrated multi-omics analysis remains challenging. Most studies to date examine each omics layer in isolation or at most in pairwise combinations. Developing methods to holistically combine genomics, epigenomics, transcriptomics, proteomics, and metabolomics data is a key frontier for future cardiovascular research.

### Genomics: genetic basis of CVDs

Genomic insights are particularly valuable for understanding MI, as they reveal genetic risk factors and mechanisms underlying the disease. Large-scale genome-wide association studies (GWAS) have identified over 200 loci linked to the risk of MI and coronary artery disease (CAD) by impacting lipid metabolism, blood pressure regulation, inflammation, and platelet function [7–12]. Notably, all genes identified—except *ABO*—were associated with risk for both MI and CAD rather than one condition independently. Recent GWAS analysis of data from approximately 639,000 individuals revealed eight new loci with significant associations with MI; six of these loci exhibited stronger associations with MI than CAD without MI [13]. For instance, the locus on chromosome 1p21.3, which includes the *SLC44A3* gene, showed a specific association with MI in the presence of CAD, rather than with coronary atherosclerosis alone, confirmed by independent replication analyses. Functional and bioinformatic studies suggest *SLC44A3* may influence plaque rupture and thrombosis directly within the vascular wall, indicated by its expression in MI-relevant vascular tissues and co-localization with atherosclerotic aorta. These findings demonstrate how GWAS can uncover novel biological targets relevant to MI.

### Transcriptomics and epigenetics: gene expression and regulation of CVDs

Transcriptomic studies provide crucial insights into the molecular response of various cell types to MI, capturing differentially expressed genes (DEGs) involved in injury and repair processes. These changes reveal acute responses that exacerbate tissue damage and highlight potential therapeutic targets. For example, single-cell RNA sequencing (scRNA-seq) has identified beta-2 microglobulin (B2M) as a key regulator of immune response and tissue repair post-MI, making it a promising therapeutic target [14].

Transcriptomic profiling has also been used to understand ventricular remodeling and systemic effects following MI. Spatial transcriptomics combined with single-nucleus RNA sequencing revealed the expression of mechanical stress response genes like *Csrp3* in the infarct border zone, underscoring their role in cardiac remodeling [15]. A genome-wide transcriptomic analysis in murine models highlighted dysregulated biological processes such as immune responses, mitochondrial dysfunction, and RNA and protein processing in tissues like the heart and liver post-MI [16]. In human studies, MI zone transcriptomics identified 1,785 DEGs, with significant upregulation of genes related to apoptosis, necroptosis, and necrosis, providing a detailed view of cell death mechanisms in infarcted tissue [17].

Epigenomics continues to add another layer of complexity to CVD research. Epigenomics examines heritable changes in gene expression that occur without alterations to the DNA sequence, focusing on mechanisms such as DNA methylation, histone modifications, and chromatin remodeling. An epigenome-wide association study (EWAS) identified 211 differentially methylated CpG sites associated with genes linked to cardiac function, CVDs, and recovery post-ischemic injury in individuals with a history of MI [18]. Additionally, nested case-control studies have mapped CpGs to genes involved in signal transduction, metabolism, and gene expression pathways, several of which are associated with MI-related conditions such as HF, stroke, and cardiac aging [19].

High-resolution integrative molecular mapping has further advanced the field. Integrating transcriptomics with epigenomics, such as DNA methylation and histone modifications, further elucidates regulatory mechanisms underlying gene expression shifts, with studies linking disruptions in these processes to pathways associated with muscle contraction and inflammation [20]. A study using single-cell gene expression, chromatin accessibility, and spatial transcriptomics in myocardium from MI patients identified distinct molecular profiles across physiological zones and time points, paving the way for novel therapeutic approaches [21]. Similarly, transcriptome and genome-wide DNA methylation analysis in acute MI mouse models revealed critical stages of differential methylation and gene expression, with the 6-h time point showing drastic changes and enriched pathways associated with the acute phase of MI. These findings were validated through in vitro studies, demonstrating regulation by DNA methylation [22]. These integrative approaches provide a comprehensive understanding of the molecular landscape in MI and HF, offering valuable biomarkers and therapeutic targets.

### Proteomics: profiling proteins in CVD research

Proteomics, the large-scale study of proteins, enables the identification of disease-associated biomarkers and the discovery of novel drug targets. By providing insights into protein structure, function, and interactions, proteomics is a powerful tool for understanding disease mechanisms and therapeutic development. In MI, proteomics has revealed key protein changes and associated pathways. A study in male Sprague-Dawley rats identified 169 differentially expressed proteins (DEPs), with 52 upregulated and 117 downregulated, linked to pathways such as complement and coagulation cascades, MAPK signaling, and actin cytoskeleton regulation [23]. Similarly, in a comparison of MI patients and those with stable CAD, 198 DEPs were identified, including proteins involved in GDP and GTP binding, coenzyme A biosynthesis, and AMPK signaling pathways [24]. Proteomics has also been instrumental in studying rare MI complications such as cardiac rupture, identifying 958 DEPs that were enriched in pathways like RNA transport, ribosome function, and necroptosis [25].

Beyond the heart, proteomics has highlighted the systemic effects of MI, including neuroinflammation. Following permanent ligation of the left anterior descending coronary artery in rats, several proteins associated with brain disease were identified in the brain cortex, including targets linked to elongation of very long chain fatty acids protein 5 (ELOVL5) and ATP-binding cassette subfamily G member 4 (ABCG4). These findings, confirmed through western blotting and immunofluorescence, underscore the interconnected nature of cardiac and neurological events [26]. Collectively, these studies demonstrate the potential of proteomics to uncover critical molecular insights into MI and its complications, paving the way for novel therapeutic strategies.

### Metabolomics: exploring metabolic changes in cardiac health

Metabolomics provides a snapshot of small molecules and metabolites within cells and tissues, offering insights into cellular energy status. The heart, composed of ATP-demanding cells, requires substantial energy to function. To sustain its activity, the adult heart metabolizes fatty acids to produce 50–70% of its ATP requirements [27, 28]. Multi-omics approaches have been instrumental in uncovering the mechanisms underlying HF. For example, proteomic and metabolomic characterization of three commonly used HF mouse models revealed downregulation of fatty acid oxidation and ATP metabolism, identifying four metabolite biomarkers associated with HF-related rehospitalization [29]. These findings underscore the potential of metabolomics to improve prognostic assessment.

Although it is well established that the heart shifts its use of carbon substrates for energy production during disease states [27, 30], the mechanisms driving these metabolic changes remain poorly understood. To address this gap, researchers developed a heart-specific genome-scale metabolic network reconstruction, enabling the interpretation of HF-related gene expression data and the identification of metabolic functions associated with these changes [31]. This approach pinpointed nitric oxide and *N*-acetylneuraminic acid as common metabolic markers of HF, offering new insights into disease progression. Together, these studies highlight the critical role of metabolomics in advancing our understanding of HF. By integrating metabolic data with other omics layers, researchers can uncover novel biomarkers and therapeutic targets, paving the way for precision medicine approaches in CVD.

## Mechanistic insights unveiled by AI through omics integration

The growth of omics datasets has led to a similar expansion of new methods to synthesize large datasets. AI is an excellent framework for synthesizing large data because it can learn complex patterns and relationships within existing datasets. AI can take multi-dimensional inputs, identify the relationships between each datapoint through its architecture, and perform classification, regression, or data compression. XAI is a type of AI that allows humans to understand and trust the results of ML algorithms. XAI addresses the limitations of traditional neural network black boxes by making AI systems’ behavior understandable to humans. It achieves this while maintaining or improving model performance, unlike black boxes that excel at accurate predictions but lack interpretability [32]. In this context, XAI is not a replacement for human intelligence, but a tool humans can employ to further their understanding of mechanisms. Between 2010 and 2023, there have been hundreds of publications that used XAI for omics data, with the top fields being medical research, oncology, clinical laboratory sciences, and neurology [5]. In this section, we will go over the XAI models that utilize omics data to output biomedical insights in oncology, neurology, and cardiology.

**Figure 3.**
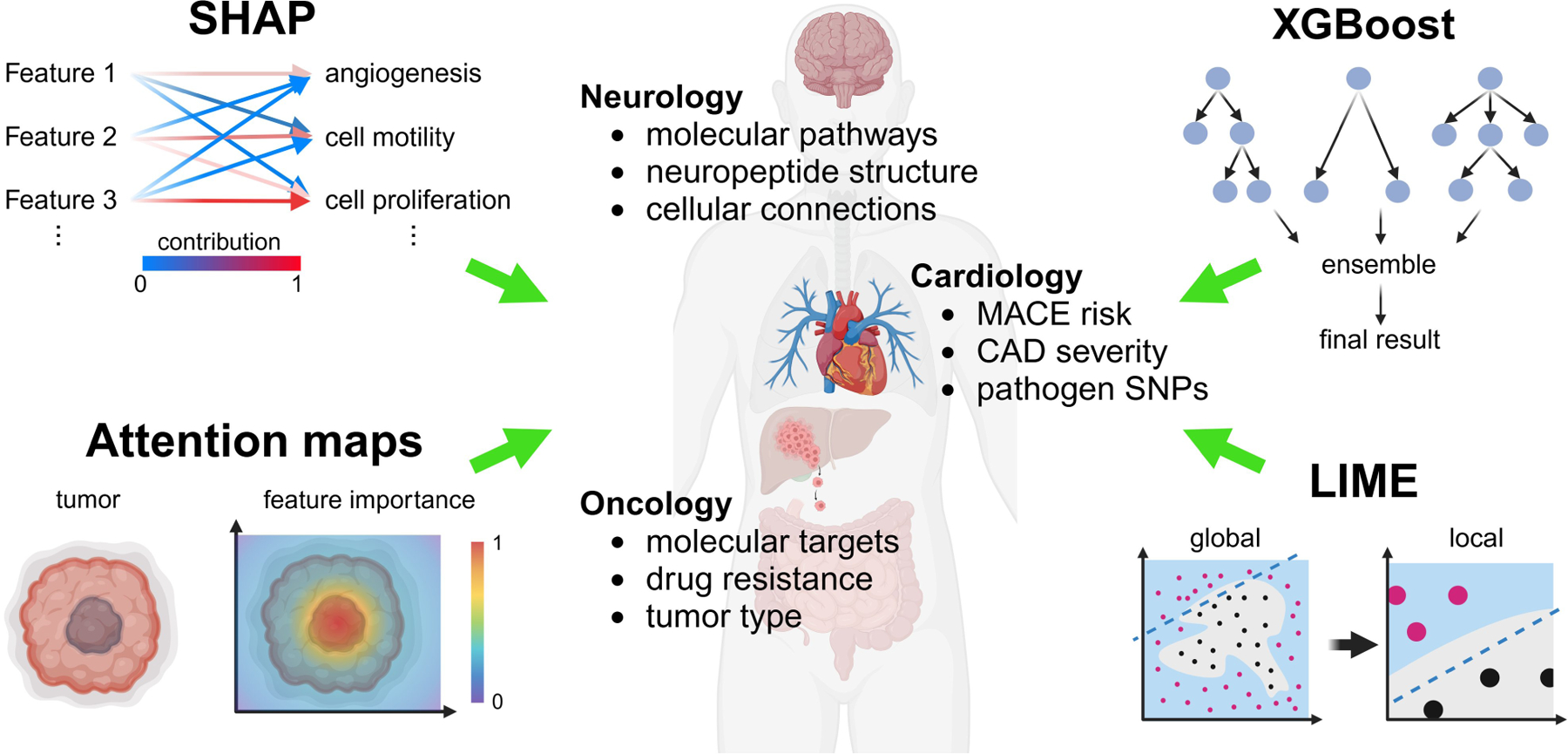
XAI-driven mechanistic insights through bioinformatics integration in biomedical research. XAI integrates multi-omics data, including genomic, transcriptomic, proteomic, and metabolomic layers, to derive insights into disease mechanisms. Visualization techniques and feature importance analyses, such as SHAP and LIME, help break down black-box machine learning models by quantifying the contribution of individual features, enabling better interpretability. XGBoost, a gradient boosting machine learning algorithm, is used to model complex relationships and predict outcomes in biomedical research, while attention maps focus on identifying important regions within input data by highlighting the most relevant features influencing the model’s decision-making. These techniques collectively highlight critical biomarkers, pathways, and molecular targets for different conditions, supporting hypothesis-driven research and the development of precision therapies. XAI: explainable artificial intelligence; SHAP: SHapley Additive exPlanations; MACE: major adverse cardiovascular events; CAD: coronary artery disease; SNPs: single nucleotide polymorphisms; XGBoost: eXtreme Gradient Boosting; LIME: Local Interpretable Model-agnostic Explanations. Created in BioRender. Sabry, Z. (2025) https://BioRender.com/u39m675

XAI techniques aim to provide transparency to complex ML models, allowing researchers to interpret and trust their predictions. These methods include feature importance analysis, visualization techniques, and surrogate models, which highlight the most influential factors contributing to a model’s decision-making process [32]. For instance, techniques like SHAP, attention maps, LIME, and eXtreme Gradient Boosting (XGBoost) break down the predictions of black-box models by quantifying the contribution of individual features (Figure 3).

Another strategy to make AI more explainable is to combine it with mechanistic models. This could involve either using the outputs from modeling as input into ML or using biological networks as the architecture for constructing neural networks. Several recent studies have used the first approach, using the outputs from metabolic models as inputs into ML methods [33–36]. This strategy allows diverse input data types to be standardized with metabolic models and provides both mechanistic interpretation and generalizability. For example, Eames and Chandrasekaran [33] used this approach to predict the depth of quiescence of mammalian cells using various data types and screened drugs that modulate quiescence. Similarly, the CARAMeL method predicts the impact of metabolic environment and heterogeneity on drug response by combining mechanistic metabolic network models with ML [34].

In contrast, the second approach—using known biological networks as a neural network’s architecture—is only beginning to be explored. In this paradigm, the layout of a neural network is designed to mirror biological pathways or the hierarchical organization of cellular processes, so that each node or layer has a defined biological meaning. A notable example is the “DCell” model, which organizes a deep neural network according to the hierarchical structure of a cell’s subsystems to predict cellular phenotypes [37]. DCell’s architecture includes modules corresponding to organelles and pathways, thereby embedding prior biological knowledge directly into the model. Although such network-in-network models have not yet been widely applied to cardiovascular data, they offer a promising avenue for ensuring that AI predictions are grounded in known biology. By constraining neural networks with actual biological network topology, this approach could enhance interpretability. For example, the CALMA approach uses a neural network architecture that mirrors the topology of metabolic networks and predicts metabolic pathways that differentially contribute to drug potency and toxicity [38]. This strategy can hence inject mechanism and physiological relevance when applied to complex multi-omics datasets in cardiology.

By offering insights into which variables, such as specific genes or metabolites, are most important for a particular prediction, XAI bridges the gap between the powerful predictive capabilities of AI and the need for biological relevance in the context of disease. This interpretability is crucial in biomedical research, where understanding the underlying biological mechanisms is as important as accurate predictions. XAI’s ability to reveal the rationale behind AI-driven insights empowers researchers to generate hypotheses, guide experimental design, and develop targeted interventions with a higher degree of confidence.

### XAI in oncology

Given the wealth of available omics data, oncology is one of the leading applications of XAI. In breast cancer, researchers have recently developed a new interpretable XAI model to classify breast cancer subtypes using three omics datasets comprising transcriptomics, DNA methylation, and microRNA expression [39]. By considering the biological relationships between the omics data, such as the inhibitory effects of DNA methylation and microRNA, the authors developed moBRCA-net, an interpretable convolutional neural network (CNN). The authors identified features, such as DEGs, associated with meaningful cross-omics layers for further analysis. CNN layers are then used to extract the high-level patterns and spatial relationships from processed omics datasets to determine breast cancer subtypes. Thus, a tool such as this can identify which molecular targets (i.e. regulators of epigenetics, transcriptomics, etc.) to develop drug candidates for based on the subtype of breast cancer.

In oncology, a large amount of bulk-RNA sequencing data has been generated examining the relationship between gene expression and drugs. However, limited data has been generated examining the relationship between single-cell transcriptomics and drugs. Researchers have recently developed the scDEAL framework, which combines two separate encoder-decoder architectures to harmonize the drug-related bulk RNA-seq data with scRNA-seq data to determine the fine-grained drug response predictions at the cellular level [40]. This process provides insights into cell-type-specific drug sensitivity and resistance, advancing approaches for drug response prediction.

To better understand the biological contribution of several features in cancer, researchers recently developed a transformer XAI that takes mutated genetic sequences and combines it with additional omics data, such as transcriptomics to determine oncology-relevant outcomes such as tumor type [41]. In the process, attention maps are employed to interpret the model’s predictions, highlighting which features, such as specific mutations or gene expression loci, contributed most to the model’s decision.

Thus, a tool such as this provides a holistic view of cancer pathology, informing researchers on relevant drug target pathways. Yet current methods only integrate a small number of omic data types, typically transcriptomics and genomics. A recent study developed the Recon8D model, that combines eight different omics networks and their interactions with metabolic networks in cancer cells [42]. This study uncovered the interplay between genetic, transcriptional, epigenetic, proteomic and post-translational regulatory networks and metabolism. It also provided insights into drug response and drug interactions across various omics layers.

### XAI in neurology

Despite having applications in practically all domains of engineering and bioinformatics, CNNs excel in handling visual tasks. Researchers have taken advantage of this by recently developing an interpretable CNN model that is trained on classifying amyotrophic lateral sclerosis (ALS) samples by analyzing images generated from RNA expression data, where RNA values are transformed into pixel representations [43]. The authors then apply SHAP to identify the most influential pixels for ALS classification. This mapping not only enables the classification of ALS, including minority classes, but also highlights genes potentially implicated in ALS molecular pathways. These insights are highly relevant to drug discovery, as identifying genes associated with ALS can inform the development of targeted therapies.

Researchers developed NeuroPpred-Fuse and NeuroPred-FRL to predict neuropeptide structure by encoding peptides as sequence-derived features which capture structural and functional properties relevant to neuropeptide prediction [44, 45]. Both models encode peptide sequences using diverse sequence-derived features, ensuring a comprehensive representation of structural and functional properties. Then, both models incorporate feature selection methods to identify the most relevant features and employ advanced ML classifiers to improve prediction accuracy. Additionally, both models emphasize interpretability by analyzing the weight contribution of selected features, facilitating a better understanding of the biological factors influencing neuropeptide prediction. These approaches collectively advance neuropeptide discovery for therapeutic applications.

Understanding the spatial distribution and heterogeneity of cells is crucial for drug discovery as it reveals how cell types interact within their microenvironment. Researchers developed ACTIONet to better understand cell diversity and interactions from single-cell transcriptomic data by clustering cells into archetypes and mapping how these archetypes relate to each other in a geometric space that reflects similarities or differences between cells [46]. The method then builds adaptable connections between cells based on their local density and relationships. For example, denser regions of cells may form tighter groups, while sparser regions allow more flexibility in how cells are linked. This adaptability ensures that the network accurately reflects the natural variation in cell populations, making it easier to explore both detailed and large-scale cell-state patterns. By applying this method to human brain data, ACTIONet was able to combine information from multiple datasets, define common cell types, and identify important genes tied to these types. This approach makes it easier to study complex tissues like the brain and potentially the heart by providing a clearer picture of cell behavior and function. This enables the identification of targeted therapies that can disrupt disease-specific cell networks or enhance tissue regeneration.

### XAI in cardiology

Cardiology has been steadily integrating AI tools over the past decade, particularly in clinical laboratory settings such as cardiovascular imaging and electrophysiology [47]. Despite the application of advanced XAI tools for understanding the molecular mechanisms in oncology and neurology, there are strikingly fewer XAI tools developed for the investigation of cardiovascular molecular mechanisms [5].

Adams et al. [48] developed an ML-based framework using Random Forest Iterative Feature Reduction and Selection (RF-IFRS) to predict the risk of major adverse cardiovascular events (MACE) in statin users by analyzing genome-wide association data. The model identified six genetic variant networks, including genes involved in vasculogenesis, angiogenesis, and vascular stability. This approach incorporates XAI by using decision tree-based visualizations to illustrate the relationships between genetic variants and their contributions to MACE risk. These insights not only predict MACE in statin users with improved accuracy but also identify biologically relevant pathways for CVD progression, offering potential targets for drug development to mitigate MACE risk and advance cardiovascular therapies.

Pezoulas et al. [49] developed a customized XGBoost model to predict carotid artery disease severity by analyzing metabolomics data from the Young Finns Study cohort. XAI is implemented through SHAP which enables researchers to understand how specific vascular and metabolic features contribute to carotid artery disease severity, represented by increased intima-media thickness. The model demonstrates high accuracy and sensitivity in detecting early carotid artery disease stages while providing valuable insights into the metabolic and structural factors underlying disease progression. By highlighting these specific factors, the model not only predicts disease severity with high accuracy but also provides actionable insights into the biological mechanisms driving the disease, which can be targeted in drug development to prevent MI and subsequent HF.

Recently, Dr. Yilmaz [50] developed a Gradient Boosted Trees (GBT) model that can distinguish between acute MI and control groups based on a metabolomics dataset. XAI is implemented through the LIME method, which highlights the contribution of specific metabolites, such as creatinine, cytosine, and myo-inositol, to the model’s predictions. This interpretability allows researchers to understand why certain metabolites are significant for classifying acute MI, enabling the identification of key biomarkers. This approach highlights the biological role of these metabolites in infarct-related metabolic processes, offering potential applications in diagnostics and therapeutic development.

Similarly, DeGroat et al. [51] used a multimodal AI/ML framework to predict CVDs by integrating transcriptomic and genomic data from a clinically integrated dataset. Using XGBoost with Bayesian optimization, the model identified transcriptomic features and pathogenic single nucleotide polymorphisms (SNPs) as predictors, achieving perfect classification in its test cohort. SHAP then provided interpretability, revealing biomarkers such as RPL36AP37 and HBA1 as the most significant contributors. Unlike Yilmaz [50], this model leveraged multi-omics data and identified not just metabolic but also genetic biomarkers. Both studies demonstrate the utility of XAI in cardiovascular biomarker discovery, paving the way for personalized treatments, though they differ in their focus on metabolomic vs. multi-omics integration and the specific cardiovascular conditions they address.

It is important to note that the above examples demonstrate that current XAI applications in cardiology typically involve one or two omics modalities, rather than fully integrated multi-omics. Of the four studies highlighted, three used a single data type (either genomics or metabolomics), and only one [51] combined two data types. This underscores that multi-omics integration in cardiovascular AI studies is still in an early phase of development. In other words, truly harmonized multi-omics analyses for heart disease are a work in progress and represent a promising area for future growth.

To summarize the state-of-the-art methods and their outcomes, Table 1 provides a brief comparison of representative XAI applications in cardiovascular research, including the data types used, AI approaches, and performance achieved.

## AI-driven drug repurposing: transforming drug discovery and development

Drug development is a lengthy, costly endeavor, with estimates suggesting that bringing a single drug from target discovery to market can cost between $314 million and $2.8 billion and often take more than a decade [52]. Such high barriers have prompted researchers to seek alternative strategies, among which drug repurposing has emerged as a particularly promising approach. Drug repurposing involves identifying new uses for existing drugs or candidates originally approved or developed for different indications. Because these agents have well-characterized safety profiles and established manufacturing processes, their development timeline and costs can be significantly reduced. This approach leverages the fact that most drugs exert multiple biological effects beyond their initially approved use. Indeed, nearly 30% of FDA-approved medications have been indicated for additional conditions beyond their original indication [53].

**Figure 4.**
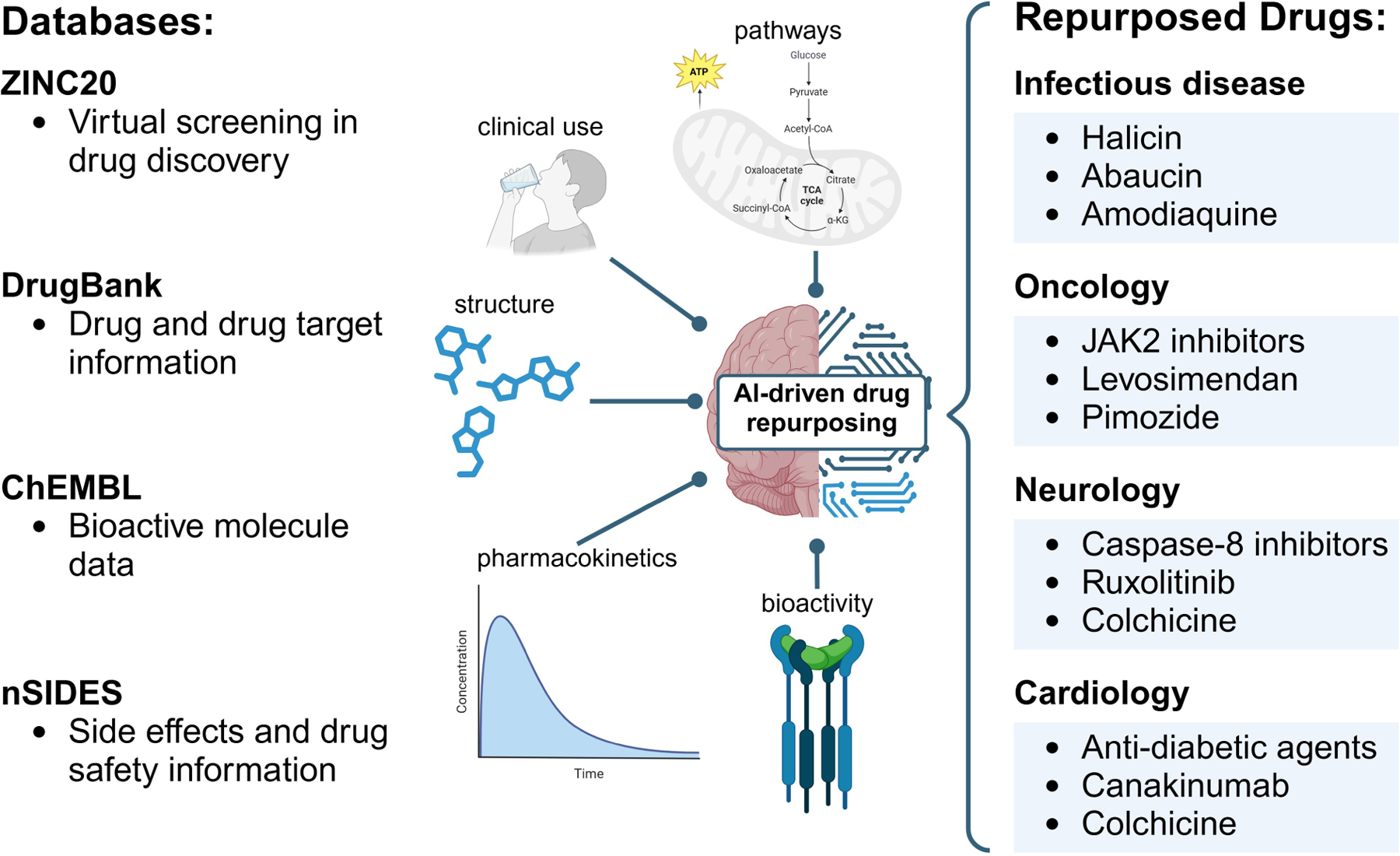
AI accelerates drug repurposing across therapeutic areas. Advances in AI and XAI methods, utilizing structural, pharmacological, and adverse effect data from databases such as DrugBank, ChEMBL, ZINC20, and nSIDES, have enabled systematic approaches to identifying repurposing candidates. For example, drugs such as JAK2 inhibitors and pimozide have been repurposed in oncology, while colchicine and canakinumab show promise in cardiology. In neurology, caspase-8 inhibitors and ruxolitinib have emerged as candidates, and in infectious diseases, drugs like halicin and abaucin have been identified. AI: artificial intelligence; XAI: explainable AI; TCA: tricarboxylic acid; CoA: coenzyme A; α-KG: alpha ketoglutarate; JAK2: Janus kinase 2. Created in BioRender. Sabry, Z. (2025) https://BioRender.com/r38i981

Historically, drug repurposing has often occurred serendipitously, such as when bupropion and gabapentin were prescribed off-label for novel uses [54, 55]. Even aspirin, first introduced as an analgesic, is now central to treatment plans for patients with cardiovascular risk [56]. While these chance discoveries have proven valuable, they are neither systematic nor sustainable. Advances in data mining and XAI now offer more deliberate, data-driven methods to identify repurposing candidates. Drugs have already been repurposed using AI in fields such as neurology, cardiology, oncology, and infectious diseases (Figure 4). These tools can analyze complex genomic and clinical datasets to reveal shared disease mechanisms or therapeutic targets, ultimately enabling a more rational and efficient repurposing process.

### AI-driven drug repurposing in oncology

Kinases are integral to numerous pathological processes, particularly in cancer, making them attractive targets for therapeutic intervention. Considerable attention has therefore been devoted to the discovery and development of small-molecule kinase inhibitors. For example, a computational platform called VirtualKinomeProfile has been used to profile compound-kinase interactions and predict the inhibitory activity of repurposed drugs, ultimately identifying 19 small-molecule inhibitors of epidermal growth factor receptor (EGFR), hematopoietic cell kinase (HCK), vascular endothelial growth factor receptor 1 (VEGFR1), and mitogen- and stress-activated kinase 1 (MSK1)—key kinases implicated in cancer progression [57]. Another ML-based effort, employing the XGBoost algorithm, identified promising Janus kinase 2 (JAK2) inhibitors from the ZINC database, of which 13 compounds underwent experimental validation and 6 demonstrated IC50 values below 100 nM [58]. Further expanding the scope, a one-drug-multi-target approach was applied to repurpose existing drugs for dual cancer indications, revealing that levosimendan, a phosphodiesterase (PDE) inhibitor effective against HF, also exhibits activity against multiple cancers, including lymphoma, through the direct inhibition of RIOK1 and other kinases [59].

Efforts in drug repurposing have not been limited to kinase inhibitors. A deep learning-driven pipeline integrating transcriptomic data and chemical structures identified pimozide—an anti-dyskinesia agent used for controlling Tourette’s disorder symptoms—as a strong candidate for treating non-small cell lung cancer [60]. Additionally, a ML-based in silico screening technique applied to FDA-approved compounds from DrugBank pinpointed candidates that inhibit the REarranged during Transfection (RET) receptor, an important factor in ligand-independent kinase activation and carcinogenesis [61]. Together, these computational strategies underscore the potential of drug repurposing, not only for identifying effective kinase inhibitors but also for revealing entirely new anticancer applications of existing therapeutics.

### AI-driven drug repurposing in neurology

Researchers have increasingly employed ML-based approaches to identify promising candidates for drug repurposing in the context of Alzheimer’s disease (AD). For instance, Rodriguez et al. [62] leveraged computational models to associate disease severity with underlying molecular mechanisms, screening 80 FDA-approved compounds to produce a ranked list of repurposing candidates. Among these, ruxolitinib, a JAK1/2 inhibitor originally developed as a targeted therapeutic for myeloproliferative neoplasms, emerged as a top hit, highlighting the potential of data-driven pipelines to pinpoint existing drugs with new therapeutic applications for AD.

Moreover, dynamic molecular descriptors derived from molecular dynamics simulations were integrated into ML models to identify compounds targeting caspase-8, a protease implicated in AD pathology [63]. Other methodologies have demonstrated similar potential. The use of graph CNNs for identifying inhibitors of the beta-secretase enzyme 1 (BACE1), a key player in amyloid-beta production, has allowed for the capture of subtle structural nuances that traditional descriptors may overlook [64]. These approaches refined candidate prioritization, ultimately guiding the selection of promising drug candidates for experimental validation.

Additional strategies have employed Bayesian ML models, combined with extended connectivity fingerprints, to systematically scan large collections of bioactive compounds—including regulatory-approved drugs available in databases like ChEMBL—for inhibitors of glycogen synthase kinase 3β (GSK3β), an enzyme central to tau phosphorylation in AD [65]. Through this process, ruboxistaurin, originally investigated for other conditions, emerged as a potent GSK3β inhibitor with an IC50 of 97.3 nM. These findings reinforce the utility of computational methods in identifying new therapeutic applications for existing drugs, streamlining the pathway to effective AD interventions.

### AI-driven drug repurposing in infectious diseases

As drug-resistant pathogens continue to emerge, both the lack of strong market incentives and diminishing returns on investment in new drug development have made drug repurposing an increasingly attractive strategy in the field of infectious diseases. For example, deep learning models trained on data from 2,335 compounds with their activity against *Escherichia coli* compounds have been used to virtually screen over 107 million molecules, leading to the discovery of halicin, a compound originally investigated for diabetes treatment, as a potent antibacterial agent [66]. Halicin demonstrated low minimum inhibitory concentrations and broad-spectrum activity, including efficacy against *Acinetobacter baumannii* in murine models. Similarly, an AI-driven screening of approximately 7,500 compounds identified abaucin as a narrow-spectrum antibacterial candidate targeting *A. baumannii*, which also effectively controlled infection in mice models [67].

In addition to identifying single-agent therapies, there is increasing interest in designing combination therapies using approved drugs to combat antibiotic resistance. Chemogenomics data has been integrated into ML frameworks to predict synergistic or antagonistic interactions of antibiotic combinations for inhibiting *E. coli* growth [68]. Transcriptomic data analysis has been employed to discover synergistic drug pairs of repurposed drugs against *Mycobacterium tuberculosis* [69]. Further refinements have accounted for temporal and sequence-dependent factors influencing drug combination effects [34, 70]. A recent study powered by a transparent ML approach screened all FDA-approved drugs for synergistic activity with existing antibiotics against *E. coli*. This study discovered the cerebrovascular drug fasudil as a potential synergizer of antibiotics and provided insights into mechanisms of synergy [71]. These advancements illustrate the growing sophistication and promise of computational strategies in optimizing combination therapy design.

A similar trend in drug repurposing emerged during the COVID-19 pandemic. Knowledge-graph-based strategies identified baricitinib, originally developed for the treatment of rheumatoid arthritis, as a candidate for treating COVID-19 by blocking JAK1, JAK2, adaptor associated kinase 1 (AAK1), and cyclin G-associated kinase [72]. In another investigation, a combination of cheminformatics and protein interaction analyses revealed that σ1 and σ2 receptor modulators, such as clemastine and cloperastine, exert antiviral activity against SARS-CoV-2 [73]. ML frameworks incorporating drug-target and protein-protein interaction data singled out ritonavir and rifampicin as potential repurposed agents for COVID-19 [74]. Moreover, virtual screening using hydroxychloroquine as a template and comparing structural similarities against nearly 4,000 approved drugs identified amodiaquine, zuclopenthixol, and nebivolol as effective in vitro agents in Vero E6 cells infected with SARS-CoV-2 [75].

Collectively, these efforts underscore the value of computational and ML methodologies in accelerating drug repurposing and combination therapy design, providing viable alternatives for managing resistant infections and emerging pathogens.

### AI-driven drug repurposing in cardiology and extrapolations

Antidiabetic agents have gained traction for their ability to lower cardiovascular mortality and MACE, underscoring a growing interest in leveraging metabolic therapies for cardiovascular benefit [76]. Beyond metabolic modulation, inflammation is increasingly recognized as a key driver of CVD, prompting investigations into the repurposing of anti-inflammatory drugs as cardioprotective agents. For instance, canakinumab, a monoclonal antibody targeting interleukin-1β initially developed for treating autoinflammatory conditions, was tested in patients with a history of MI and elevated C-reactive protein levels [77]. After 48 months of treatment, reductions in inflammatory biomarkers and improved cardiovascular outcomes were observed, demonstrating the promise of an anti-inflammatory approach. Similar findings were reported for colchicine, a drug traditionally used for gout attacks. In individuals with chronic coronary disease, treatment with colchicine led to a significant decrease in cardiovascular events, and when administered shortly after MI, reduced the risk of subsequent ischemic events [78, 79].

Collectively, these studies underscore the potential of repurposed anti-inflammatory agents for addressing the inflammatory underpinnings of CVD. Although AI approaches have demonstrated efficacy in other therapeutic areas, including neurology and oncology, their application in cardiovascular drug repurposing is only beginning to emerge. By integrating ML algorithms with large-scale clinical, genomic, and proteomic data, AI-driven frameworks could efficiently uncover novel drug-disease associations, predict therapeutic responses, and refine dosing strategies. Such capabilities promise to streamline the drug development pipeline, reducing both time and cost. As research continues to elucidate the intricate interplay between metabolic and inflammatory pathways in CVD, leveraging AI-enhanced bioinformatics for drug repurposing may catalyze the translation of preclinical insights into safe, effective cardiovascular therapies.

## Challenges and future directions

Despite significant advances in the use of AI and XAI for biomedical research, several critical challenges continue to hinder their widespread adoption in cardiovascular drug discovery. One foremost limitation is the heterogeneity of data. MI and HF are clinically diverse conditions, encompassing multiple subtypes—for example—ST-segment elevation MI (STEMI) vs. Non STEMI (NSTEMI) or HF with preserved ejection fraction (HFpEF) vs. reduced ejection fraction (HFrEF). Combining such heterogeneous data during model training can obscure meaningful biological patterns and reduce predictive performance. To address this, future research should employ more stringent classification schemes—such as the Universal Definition of MI (UDMI) or established HF staging frameworks—to create more homogeneous datasets that facilitate better model accuracy and interpretability.

Another significant challenge is the limited integration of multi-omics data. While individual omics layers—such as genomics, transcriptomics, or metabolomics—have been used successfully in various studies, few models can fully harmonize multiple data modalities in a biologically meaningful way. Integrated multi-omics models remain scarce due to technical difficulties, such as discrepancies in data scale, batch effects, missing values across datasets, and lack of matched multi-omic measurements performed on the same patient or tissue samples. Furthermore, establishing standardized pipelines for omics data harmonization and fusion will be essential to progress in this area.

Compounding these issues is the broader challenge of sustaining AI frameworks and ensuring their long-term usability. Many bio-inspired AI tools lack continued support, regular maintenance, or active community engagement, which can limit reproducibility and hinder adoption. A notable example is DCell, a deep learning framework designed to simulate cell responses to genetic perturbations using structured biological knowledge. However, the original DCell implementation appears to be no longer actively maintained. Online resources, including code and documentation, are outdated, which may hinder reproducibility and practical application of DCell. The deprecation of DCell highlights a broader challenge in the sustainability of bio-inspired AI frameworks: the lack of long-term support, maintenance, and community adoption. To address this, the field should prioritize open-source development with clear documentation, modular codebases, and active community engagement to ensure usability beyond initial publication. Establishing centralized repositories, incentivizing reproducibility through funding and publication standards, and promoting interoperability with emerging datasets and platforms can help maintain relevance.

The issue of model bias and transparency is also paramount. AI models often inherit biases present in training data, leading to skewed predictions that can disproportionately affect underrepresented populations or reinforce known research biases. While XAI tools such as SHAP and LIME help to mitigate these issues by making model decisions interpretable, these tools are still evolving and may not fully resolve all transparency concerns. To enhance trust and usability, future models should incorporate fairness-aware training algorithms and adhere to transparent reporting practices, including clear documentation of data provenance, model assumptions, and limitations.

Finally, the field lacks standardized benchmarks for evaluating the performance and utility of different AI/XAI approaches in drug discovery. Without community-agreed evaluation metrics or gold-standard datasets, comparing models and validating results is challenging. Therefore, establishing public benchmarks and fostering a culture of open science—where datasets, code, and model parameters are freely shared—will be essential for driving reproducibility and innovation.

## Conclusions

Despite CVD being the leading cause of mortality worldwide and the availability of extensive omics datasets, the application of XAI to elucidate the molecular mechanisms of heart disease remains scarce compared to fields like oncology and neurology. A recent systematic mapping study of XAI literature in biomedical research identified only 5 out of 405 publications (2010–2023) as cardiology-focused [30]. This gap persists despite the well-recognized difficulties in developing effective cardiovascular drugs—a process hindered by the complexity and heterogeneity of CVDs and the stringent efficacy and safety criteria required for regulatory approval. Additionally, large-scale clinical trials, often essential for assessing long-term outcomes such as reductions in cardiovascular mortality and MACE, further inflate both costs and development timelines.

Under these circumstances, integrating XAI methods with large-scale, multi-omics datasets could be instrumental in deciphering therapeutic molecular mechanisms and guiding rational drug repurposing strategies for CVDs. Moreover, as the drug repurposing landscape evolves, computational methods and AI-driven platforms are increasingly central to accelerating drug discovery. Through the integration of clinical, genomic, and molecular data, ML algorithms can identify promising drug targets, predict therapeutic responses, and streamline candidate selection. Beyond improving efficiency and reducing trial-and-error, these tools may uncover unexpected repurposing opportunities.

While XAI-based drug discovery holds enormous potential, several challenges remain that need to be addressed. For example, existing methods accelerate only the early part of the drug discovery process. New methods need to be developed to reduce toxicity, improve metabolism, and increase the selectivity of new treatments [80]. Further, AI methods may reinforce existing biases as certain populations, diseases, and proteins are more well-studied than others. Making the AI models more interpretable and interrogating them for biases may reduce these issues [81, 82]. Ultimately, the synergy between XAI, AI-driven data analytics, and repurposing strategies holds the potential to hasten the discovery of effective anti-inflammatory agents, metabolic modulators, and other innovative therapies, ushering in a more efficient, personalized era of cardiovascular medicine.

## Figures and Tables

**Table 1.** Summary of representative XAI applications in cardiovascular research, highlighting the data types used, AI methods, and performance outcomes

Study (year)	Data & cohort	AI method	XAI method	Task/Outcome	Performance
Adams et al. [48] (2020)	Genomic (GWAS of statin-treated patients, *n* = 5,890)	Random forest (RF-IFRS)	Decision tree-based network visualization	Predict risk of on-statin MACE	No standard metric reported (identified 6 variant networks; highest OR ~4.5 for the high-risk subgroup)
Pezoulas et al. [49] (2022)	Metabolomics (Young Finns cohort)	XGBoost (hybrid ML model)	SHAP (global feature importance)	Predict carotid artery disease severity (intima-media thickness)	High accuracy and sensitivity (~90% accuracy reported)
Yilmaz [50] (2023)	Metabolomics (serum metabolites in acute MI patients vs. controls)	GBT	LIME (local explanation)	Classify acute MI vs. healthy controls	High classification accuracy (effective separation of MI vs. control samples)
DeGroat et al. [51] (2024)	Multi-omics (mRNA expression + SNPs; CVD patients vs. controls)	XGBoost (with Bayesian tuning)	SHAP (feature importance)	Classify CVD patients vs. controls; identify key biomarkers	100% accuracy on the test set (*n* = 15; AUC = 1.00)

AI: artificial intelligence; XAI: explainable AI; GWAS: genome-wide association studies; RF-IFRS: Random Forest Iterative Feature Reduction and Selection; MACE: major adverse cardiovascular events; XGBoost: eXtreme Gradient Boosting; ML: machine learning; SHAP: SHapley Additive exPlanations; MI: myocardial infarction; GBT: Gradient Boosted Trees; LIME: Local Interpretable Model-agnostic Explanations; SNPs: single nucleotide polymorphisms; CVD: cardiovascular disease; OR: odds ratio; AUC: area under the curve

## Abbreviations

AD: Alzheimer’s disease
AI: artificial intelligence
ALS: amyotrophic lateral sclerosis
CAD: coronary artery disease
CNN: convolutional neural network
CVDs: cardiovascular diseases
DEGs: differentially expressed genes
DEPs: differentially expressed proteins
*E. coli*: Escherichia coli
GWAS: genome-wide association studies
HF: heart failure
JAK2: Janus kinase 2
LIME: Local Interpretable Model-agnostic Explanations
MACE: major adverse cardiovascular events
MI: myocardial infarction
ML: machine learning
SHAP: SHapley Additive exPlanations
XAI: explainable artificial intelligence
XGBoost: eXtreme Gradient Boosting
